# Rational maps of balls and their associated groups

**DOI:** 10.1007/s40863-024-00430-x

**Published:** 2024-05-14

**Authors:** Dusty Grundmeier, Jiří Lebl

**Affiliations:** 1 Department of Mathematics, The Ohio State University, Columbus, OH 43210 USA; 2 Department of Mathematics, Oklahoma State University, Stillwater, OK 74078 USA

**Keywords:** Proper, Holomorphic maps, Rational maps, Automorphism groups, Primary 32H35, Secondary 32H02, 32M99, 32M05

## Abstract

Given a proper, rational map of balls, D’Angelo and Xiao introduced five natural groups encoding properties of the map. We study these groups using a recently discovered normal form for rational maps of balls. Using this normal form, we also provide several new groups associated to the map.

## Introduction

Suppose $f :{{\mathbb {B}}}_n \rightarrow {{\mathbb {B}}}_N$ is a rational proper map, $f = \frac{p}{g}$ where $p :{{\mathbb {C}}}^n \rightarrow {{\mathbb {C}}}^N$ and $g :{{\mathbb {C}}}^n \rightarrow {{\mathbb {C}}}$ are polynomials, $\frac{p}{g}$ is in lowest terms, and $g(0) = 1$. Further, suppose *f* is of degree *d*. We say that *f* is in *normal form* if $f(0) = p(0) = 0$,

$$\begin{aligned} g = 1 + g_2 + \cdots + g_{d-1} \quad \text {and} \quad g_2(z) = \sum _{\ell =1}^n \sigma _\ell z_\ell ^2 \quad 0 \le \sigma _1 \le \cdots \le \sigma _n , \end{aligned}$$

where $g_k$ are homogeneous polynomials of degree *k*. That is, *f* is in normal form if it fixes the origin, the denominator has no linear terms, and the quadratic terms are diagonalized. In [1] the second author proved that every rational proper map of balls can be put into the normal form above via spherical equivalence and that this is a normal form up to unitary automorphisms where the source automorphism fixes $g_2$.

A natural problem is to study holomorphic maps invariant under subgroups of the automorphism group. This problem has a long history, going back to at least Cartan in [2]. We refer the reader to [3–11] and especially D’Angelo’s books [12–14]. Rudin [11] and Forstnerič [8] studied invariant proper maps to general domains in ${\mathbb {C}}^n$. In this context, one can construct proper maps invariant under any finite subgroup of the unitary group *U*(*n*). On the other hand, if the target domain is required to be a unit ball, then the problem has more structure. Forstnerič [15] showed that if the map is sufficiently smooth up to the boundary, then the map must be rational, and furthermore, in [9], he showed that for most groups, it is not possible to find a proper, rational map of balls invariant under that group. In [7], D’Angelo and Lichtblau gave the decisive result, completely characterizing which groups admit invariant, proper, rational maps between balls.

Moreover, given a proper map of balls, one can use various groups to detect various properties of the map. In particular, D’Angelo and Xiao [3, 4] introduced the groups $A_f$, $G_f$, $\Gamma _f$, $T_f$, and $H_f$, defined below. Using the normal form, we add several new groups, $D_f$, $\Sigma _f$, $\Delta ^{(a,b)}_f$.

### Definition 1.1

Suppose $f :{{\mathbb {B}}}_n \rightarrow {{\mathbb {B}}}_N$ is a rational proper map.

(i) $A_f$ is the subgroup of ${\text {Aut}}({{\mathbb {B}}}_n) \oplus {\text {Aut}}({{\mathbb {B}}}_N)$ such that $(\varphi ,\tau ) \in A_f$ if $\tau \circ f = f \circ \varphi$.
(ii) $\Gamma _f$ is the subgroup of ${\text {Aut}}({{\mathbb {B}}}_n)$ such that $\varphi \in \Gamma _f$ if there is a $\tau \in {\text {Aut}}({{\mathbb {B}}}_N)$ such that $\tau \circ f = f \circ \varphi$.
(iii) $G_f$ is the subgroup of ${\text {Aut}}({{\mathbb {B}}}_n)$ such that $\varphi \in G_f$ if $f = f \circ \varphi$.
(iv) $T_f$ is the subgroup of ${\text {Aut}}({{\mathbb {B}}}_N)$ such that $\varphi \in T_f$ if there is a $\varphi \in {\text {Aut}}({{\mathbb {B}}}_n)$ such that $\tau \circ f = f \circ \varphi$.
(v) $H_f$ is the subgroup of ${\text {Aut}}({{\mathbb {B}}}_N)$ such that $\tau \in H_f$ if $\tau \circ f = f$.

Given a polynomial $\rho (z, {\bar{z}})$, let $\rho _{(a,b)}$ denote the monomials of $\rho$ that are of degree *a* in the holomorphic *z* variables, and degree *b* in the antiholomorphic ${\bar{z}}$ variables. We refer $\rho _{(a,b)}$ as the bidegree-(*a*, *b*) part of $\rho$, and we write $*$ instead of *a* or *b* to denote all degrees together.

### Definition 1.2

Let $f=\frac{p}{g}:{{\mathbb {B}}}_n \rightarrow {{\mathbb {B}}}_N$ be a proper, rational map in normal form.

(i) $D_f$ is the subgroup of *U*(*n*) such that $U \in D_f$ when $g \circ U = g$.
(ii) $\Sigma _f$ is the subgroup of *U*(*n*) such that $U \in \Sigma _f$ when $g_2 \circ U = g_2$.
(iii) $\Delta ^{(a,b)}_f$ is the subgroup of *U*(*n*) such that $U \in \Delta ^{(a,b)}_f$ when
  $${\begin{aligned} \bigl (|{g(z)} |^2-\lVert{p(z)} \rVert^2\bigr )_{(a,b)} = \bigl (|{g(Uz)} |^2-\lVert{p(Uz)} \rVert^2\bigr )_{(a,b)}. \end{aligned}}$$

All the groups are closed, and therefore Lie subgroups. D’Angelo-Xiao [4] showed this for the groups $A_f,\Gamma _f,G_f,T_f,H_f$, and it is immediate for the new groups we defined. As long as they are compact, it follows from standard theory that they can be conjugated to a subgroup of the unitary group. In fact, $\Gamma _f$ is noncompact if and only if *f* is linear fractional (an automorphism if $n=N$), see [4]. Moreover, Lichtblau [16] (see also D’Angelo-Lichtblau [7]) has shown that $G_f$ must be finite, fixed-point-free, and cyclic. In the present paper, we prove is that once the map is in normal form, the relevant groups are all subgroups of the unitary group. Note that if *f* is in normal form and it is linear fractional, then it is in fact linear.

### Theorem 1.3

Suppose $f :{{\mathbb {B}}}_n \rightarrow {{\mathbb {B}}}_N$ is a rational proper map in normal form and *f* is not linear. Then

(i) $A_f \le U(n) \oplus U(N)$ is a closed subgroup.
(ii) $G_f \le \Gamma _f \le D_f \le \Sigma _f \le U(n)$ and $\Gamma _f \le \Delta ^{(a,b)}_f$ are all closed subgroups.
(iii) $H_f \le U(N)$ and $T_f \le U(N)$ are closed subgroups.

In particular, for a mapping that is not an automorphism, once the mapping is in normal form, all the groups are subgroups of the unitary group. One motivation for introducing the groups $\Sigma _f$, $D_f$, and $\Delta ^{(a,b)}_f$ is that they are often easier to compute. If the $\sigma$ invariants are nonzero and distinct, namely, $0< \sigma _1< \ldots < \sigma _n$, then $\Sigma _f \cong ({{\mathbb {Z}}}_2)^n$. Therefore, a corollary is that $G_f$ must be cyclic and fixed-point-free for such *f*. In general, standard linear algebra says that $\Sigma _f$ is a direct sum of groups where if we have *k* zero $\sigma$’s, the first factor is *U*(*k*), and for each set of *k* nonzero equal $\sigma$s we get a factor of *O*(*k*) (real orthogonal group). In particular, when all $\sigma$s are nonzero and distinct we get a direct sum of $O(1)=\{1,-1\}$.

### Corollary 1.4

Suppose $f = \frac{p}{g} :{{\mathbb {B}}}_n \rightarrow {{\mathbb {B}}}_N$ is a rational proper map in normal form such that $0< \sigma _1< \ldots < \sigma _n$. Then $G_f$ is either trivial, or $G_f = \{ I, -I \}$. In particular, if $G_f = \{ I, -I \}$, then $p(z) = p(-z)$ and $g(z)=g(-z)$, and the degree of *f* is at least 4.

Furthermore, for any sufficiently small $0< \sigma _1< \ldots < \sigma _n$, there exists a degree 4 map $f = \frac{p}{g} :{{\mathbb {B}}}_n \rightarrow {{\mathbb {B}}}_N$ where $g(z) = 1+\sigma _1 z_1^2 + \cdots + \sigma _n z_n^2$ and such that $G_f = \{ I, -I \}$.

In particular, the corollary says that when $\sigma _1, \ldots , \sigma _n$ are nonzero and distinct, then for $G_f$ to be nontrivial, the map *f* must have degree at least 4. Hence, when the degree of *f* is 3, we have that $G_f$ is trivial, but we then note that $D_f = \Sigma _f \cong ({{\mathbb {Z}}}_2)^n$. It is not difficult to find a map of degree 4 or higher with a denominator of the form

1 $$\begin{aligned} 1+\sigma _1 z_1^2 + \cdots + \sigma _n z_n^2 + \epsilon _1 z_1^3+\cdots +\epsilon _n z_n^3 \end{aligned}$$

for small enough $\sigma$’s and $\epsilon$’s (see Proposition 5.1 for example), in which case $D_f = \{ I \}$.

A natural problem is to determine what properties of the map *f* can be detected using the above groups. In particular, we focus on $\Gamma _f$. D’Angelo and Xiao [3] proved that a rational proper map of balls whose $\Gamma _f$ group is not compact is spherically equivalent to the linear embedding and therefore is linear fractional, in which case $\Gamma _f = {\text {Aut}}({{\mathbb {B}}}_n)$. Gevorgyan, Wang, and Zimmer [17] extended this result to maps that are only $C^2$ up to the boundary. D’Angelo and Xiao [3] further proved that $\Gamma _f$ contains the torus if and only if *f* is spherically equivalent to a monomial map (a map where each component is a single monomial). They also prove that if the group $\Gamma _f$ contains the center of *U*(*n*) (the circle group $\{ e^{i\theta } I \}$), then the map *f* is spherically equivalent to a polynomial map. They also show that for any finite subgroup $\Gamma$, there exists a rational *f* such that $\Gamma _f = \Gamma$. We give a slightly stronger version of this result in the next theorem. We say a subgroup $\Gamma \le U(n)$ is defined by finitely many invariant polynomials if there exists polynomials $\rho _1,\ldots , \rho _\ell$ such that

$$\begin{aligned} \Gamma = \bigl \{ U \in U(n): \rho _j(Uz,{\overline{Uz}}) = \rho _j(z,{\bar{z}}), j=1,\ldots , \ell \bigr \}. \end{aligned}$$

### Theorem 1.5

Suppose that $f :{{\mathbb {B}}}_n \rightarrow {{\mathbb {B}}}_N$ is a rational proper map in normal form and *f* is not linear. Then $\Gamma _f \le U(n)$ is a subgroup that is defined by a single invariant polynomial.

Conversely, given any group $\Gamma \le U(n)$ that is defined by finitely many invariant polynomials, there exists a rational, proper map $f :{{\mathbb {B}}}_n \rightarrow {{\mathbb {B}}}_N$ such that $\Gamma _f = \Gamma$. Moreover, this map *f* can be chosen to be a polynomial that takes the origin to origin, and hence in normal form.

We conclude the introduction by outlining the results of the paper. In Sect. 2, we briefly recall the usual background on Hermitian forms. In Sect. 3, we give a sequence of lemmas to prove Theorem 1.3. In Sect. 4, we prove Theorem 1.5 and characterize the possible $\Gamma _f$ groups. In Sect. 5, we consider the problem of constructing maps with a given denominator. Finally, in Sect. 6, we prove Corollary, 1.4 and give an example.

## Hermitian forms

In this section, we briefly recall the standard setup to treat real-valued polynomials as Hermitian forms (see Chapter 1 of [12] for more details on this approach). A real-valued polynomial in ${{\mathbb {C}}}^n$ can be written as a polynomial

2 $$\begin{aligned} r(z,{\bar{z}}) = \sum _{\alpha \beta } c_{\alpha \beta } z^\alpha {\bar{z}}^\beta , \end{aligned}$$

where $\alpha$ and $\beta$ are multiindices. The coefficients $c_{\alpha \beta }$ can be put into a matrix $[c_{\alpha \beta }]_{\alpha ,\beta }$ by putting an order on the monomials (and hence the multiindices) and having $\alpha$ refer to rows and $\beta$ to columns. The matrix $[c_{\alpha \beta }]_{\alpha ,\beta }$ is called the *matrix of coefficients* of *r*. The polynomial *r* is real-valued if and only if the matrix $[c_{\alpha \beta }]_{\alpha ,\beta }$ is Hermitan. Diagonalizing the matrix of coefficients then yields

3 $$\begin{aligned} r(z,{\bar{z}}) = \Vert {P(z)} \Vert ^2-\Vert {G(z)} \Vert ^2 \end{aligned}$$

where $\Vert {\cdot } \Vert$ denotes the standard Hermitian norm, and $P :{{\mathbb {C}}}^n \rightarrow {{\mathbb {C}}}^a$ and $G :{{\mathbb {C}}}^n \rightarrow {{\mathbb {C}}}^b$ are polynomials whose components are linearly independent. As the rank is $a+b$, this expansion cannot be done with fewer than $a+b$ polynomials. In particular, if the matrix of coefficients of *r* is positive semidefinite, then $r(z,{\bar{z}}) = \Vert {P(z)} \Vert ^2$, and conversely, if *r* is a Hermitian sum of squares, its matrix is positive semidefinite. The polynomial *P* in $r(z,{\bar{z}})=\Vert {P(z)} \Vert ^2$ only needs to use those monomials that correspond to nonzero rows or columns of the matrix. Hence, if the entries in the matrix corresponding to purely holomorphic or purely antiholomorphic terms are all zero, then this corresponds to the row and column corresponding to the monomial 1. In other words, in this case, *P* can be chosen to have no constant term, that is, $P(0)=0$.

## Groups are subgroups of the unitaries

Given a proper rational map $\frac{p}{g} :{{\mathbb {B}}}_n \rightarrow {{\mathbb {B}}}_N$, consider its *underlying form*

4 $$\begin{aligned} r(z,{\bar{z}}) = |{g(z)} |^2-\Vert {p(z)} \Vert ^2 . \end{aligned}$$

Since rescaling *r* by a positive constant does not change the corresponding map, we will generally normalize *r* so that $r(0,0)=1$. The following observation was made in [18], and also used later by D’Angelo-Xiao [3]. That is, two maps differ by a target automorphism if and only if the underlying forms are the same (up to rescaling).

### Lemma 3.1

(Lemma 2.1 from [1]) Suppose $\frac{p}{g} :{{\mathbb {B}}}_n \rightarrow {{\mathbb {B}}}_N$ and $\frac{P}{G} :{{\mathbb {B}}}_n \rightarrow {{\mathbb {B}}}_N$ are proper rational maps written in lowest terms such that $|{g(0)} |^2-\Vert {p(0)} \Vert ^2 = 1$ and $|{G(0)} |^2-\Vert {P(0)} \Vert ^2 = 1$. Then there exists a $\tau \in {\text {Aut}}({{\mathbb {B}}}_N)$ such that

5 $$\begin{aligned} \tau \circ \frac{p}{g} = \frac{P}{G} \qquad \text {if and only if} \qquad |{g(z)} |^2-\Vert {p(z)} \Vert ^2 = |{G(z)} |^2-\Vert {P(z)} \Vert ^2 . \end{aligned}$$

The lemma says that the group $\Gamma _f$ is precisely the group that leaves the underlying form unchanged up to rescaling. We prove Theorem 1.3 in stages using the following lemmas.

We briefly recall some properties of automorphisms and the normal form from [1]. Let $\alpha \in {{\mathbb {B}}}^n$ and $t=\sqrt{1-\Vert {\alpha } \Vert ^2}$. Then every automorphism of ${{\mathbb {B}}}_n$ is of the form $U\varphi _{\alpha }$ where $U \in U(n)$ and

6 $$\begin{aligned} \varphi _{\alpha }(z)=\frac{\alpha -L_{\alpha }z}{1-\langle z, \alpha \rangle }, \quad \text { and }\quad L_\alpha z= \frac{\langle z, \alpha \rangle }{t+1}\alpha + t z. \end{aligned}$$

See Chapter 1 of [12] for background on automorphisms of ${{\mathbb {B}}}_n$. Now, we define the $\Lambda _f: {{\mathbb {B}}}_n \rightarrow {{\mathbb {R}}}$ function by

7 $$\begin{aligned} \Lambda _f(z,{\bar{z}}) = \frac{|{g(z)} |^2-\Vert {p(z)} \Vert ^2}{(1-\Vert {z} \Vert ^2)^d} = \frac{r(z,{\bar{z}})}{(1-\Vert {z} \Vert ^2)^d}. \end{aligned}$$

In [1], the second author proves the following properties of the $\Lambda _f$ function.

### Lemma 3.2

(From [1]) If $f=\frac{p}{g}: {{\mathbb {B}}}_n \rightarrow {{\mathbb {B}}}_N$ is a rational proper map of degree $d>1$, in lowest terms, then the following hold.

1. If $$\tau \in {\text {Aut}}({{\mathbb {B}}}_N)$$, then $$\Lambda _f=\Lambda _{\tau \circ f}$$.
2. If $$\psi \in {\text {Aut}}({{\mathbb {B}}}_n)$$, then $$\Lambda _{f} \circ \psi = C \Lambda _{f \circ \psi }$$.
3. The $\Lambda$-function has a unique critical point in ${{\mathbb {B}}}_n$.
4. There exists $\tau \in {\text {Aut}}({{\mathbb {B}}}_N)$ such that $\tau \circ f \circ \varphi _\alpha$ takes the origin to the origin and has no linear terms in its denominator when written in lowest terms if and only if $\alpha \in {{\mathbb {B}}}_n$ is the critical point of the corresponding $\Lambda$-function.

### Lemma 3.3

Suppose $f :{{\mathbb {B}}}_n \rightarrow {{\mathbb {B}}}_N$ is a rational proper map in normal form and *f* is not linear. Then $A_f \le U(n) \oplus U(N)$ is a closed subgroup.

### Proof

Suppose $f = \frac{p}{g}$ is a rational proper map in normal form that is not linear and let $r(z,{\bar{z}}) = |{g(z)} |^2-\Vert {f(z)} \Vert ^2$. Suppose that $(\varphi ,\tau ) \in A_f$, thus $\tau \circ f \circ \varphi ^{-1} = f$. Write $f \circ \varphi ^{-1} = F(z) = \frac{P(z)}{G(z)}$ and let ${\tilde{r}}(z,{\bar{z}}) = |{G(z)} |^2-\Vert {P(z)} \Vert ^2$. Assume that ${\tilde{r}}(0,0) = 1$. By Lemma 3.1, we have that ${\tilde{r}}(z,{\bar{z}}) = r(z,{\bar{z}})$. Since *f* of degree strictly greater than 1, Lemma 3.2 shows $\Lambda _f$ has a unique critical point. Since ${\tilde{r}} = r$, we find that $\Lambda _f= \Lambda _F$. As *f* is in normal form, the critical point of $\Lambda _f$ is at the origin. Again from the previous lemma, $\Lambda _F = \Lambda _{f \circ \varphi ^{-1}} = C \Lambda _f \circ \varphi ^{-1}$ for some constant *C*. But this means that $\varphi$ fixes this critical point, that is, $\varphi (0)=0$. An automorphism of the unit ball fixing the origin must be a unitary map (Corollary 1.6 of [12]), and hence $\varphi \in U(n)$. Since *f* is in normal form, fixes the origin, and $\tau \circ f \circ \varphi ^{-1} = f$, we find that $\tau$ also fixes the origin and that $\tau \in U(N)$.

That $A_f$ is closed already follows from D’Angelo and Xiao [3, 4], and it is also an immediate consequence of $A_f \le U(n) \oplus U(N)$. $\square$

In particular, the lemma above gives $\Gamma _f \le U(n)$, but we can read even more from the proof. We thus have the following characterization of $\Gamma _f$, where no rescaling is necessary.

### Lemma 3.4

Suppose $f = \frac{p}{g} :{{\mathbb {B}}}_n \rightarrow {{\mathbb {B}}}_N$ is a proper rational map in normal form that is not linear. Then $U \in \Gamma _f \le U(n)$ if and only if

8 $$\begin{aligned} |{g(z)} |^2-\Vert {p(z)} \Vert ^2 \equiv |{g(Uz)} |^2-\Vert {p(Uz)} \Vert ^2 . \end{aligned}$$

In other words, $\Delta ^{(*,*)}_f = \Gamma _f$. Moreover, $\Gamma _f \le \Delta ^{(a,b)}_f$.

### Proof

That $\Gamma _f \le U(n)$ follows from the lemma above. If $U \in \Gamma _f$, then there is a $V \in U(N)$ such that $V \circ f \circ U = f$. Plugging into the underlying form and clearing denominators obtains

9 $$\begin{aligned} |{g(U z)} |^2-\Vert {p(U z)} \Vert ^2 = |{g(U z)} |^2-\Vert {V p(U z)} \Vert ^2 = |{g(z)} |^2-\Vert {p(z)} \Vert ^2 . \end{aligned}$$

Conversely, suppose $|{g(z)} |^2-\Vert {p(z)} \Vert ^2 \equiv |{g(Uz)} |^2-\Vert {p(Uz)} \Vert ^2$. Then Lemma 3.1 says that $f = \psi \circ f \circ U$ for some automorphism $\psi$, which is a unitary as $A_f \le U(n) \oplus U(N)$. $\square$

### Lemma 3.5

Suppose $f :{{\mathbb {B}}}_n \rightarrow {{\mathbb {B}}}_N$ is a proper rational map of degree $d > 1$. Then $\Delta ^{(2,0)}_f = \Sigma _f$ and $\Delta ^{(*,0)}_f = D_f$.

### Proof

First, put $f = \frac{p}{g}$ into normal form where $g(0)=1$. Complexify $|{g(z)} |^2-\Vert {p(z)} \Vert ^2$, and note that if $U \in \Delta ^{(*,0)}_f$ then

10 $$\begin{aligned} g(Uz){\bar{g}}({\overline{Uz}})-p(Uz) \cdot {\bar{p}}({\overline{Uz}}) = g(z){\bar{g}}({\bar{z}})-p(z) \cdot {\bar{p}}({\bar{z}}) . \end{aligned}$$

Now plug in ${\bar{z}}=0$ to find

11 $$\begin{aligned} g(Uz) = g(Uz){\bar{g}}(0)-p(Uz) \cdot {\bar{p}}(0) = g(z){\bar{g}}(0)-p(z) \cdot {\bar{p}}(0) = g(z) . \end{aligned}$$

Hence $U \in D_f$. Next we note that

12 $$\begin{aligned} \bigl (|{g(z)} |^2-\Vert {p(z)} \Vert ^2\bigr )_{(*,0)} = g(z){\bar{g}}(0)-p(z) \cdot {\bar{p}}(0) = g(z) , \end{aligned}$$

which implies that if $U \in \Delta ^{(*,0)}_f$, then $U \in D_f$.

The statement for $\Sigma _f$ follows analogously by considering only the quadratic terms. $\square$

### Lemma 3.6

Suppose $f :{{\mathbb {B}}}_n \rightarrow {{\mathbb {B}}}_N$ is a rational proper map in normal form and *f* is not linear. Then

(i) $G_f \le \Gamma _f \le D_f \le \Sigma _f \le U(n)$ and $\Gamma _f \le \Delta ^{(a,b)}_f$ are all closed subgroups.
(ii) $H_f \le U(N)$ and $T_f \le U(N)$ are closed subgroups.

### Proof

The containment in the unitary follows since $A_f \le U(n) \oplus U(N)$. That $A_f$, $\Gamma _f$, $G_f$, $T_f$, and $H_f$ are closed was proved by D’Angelo and Xiao in their work, and it also follows rather quickly once they are subgroups of the unitary group. That $D_f$, $\Sigma _f$, $\Delta ^{(a,b)}_f$ are closed follows as they are given by an invariant polynomial. The groups $\Gamma _f$, $G_f$, $T_f$ and $H_f$ are either equivalent to subgroups of $A_f$ or the projections onto the first or the second factor. In either case, it follows that they are all subgroups of the correct unitary groups.

The inclusions $G_f \le \Gamma _f$, $\Gamma _f \le \Delta ^{(a,b)}_f$, and $D_f \le \Sigma _f$ follow immediately. That $\Gamma _f \le D_f$ follows from Lemma 3.5. $\square$

Theorem 1.3 follows from the lemmas above.

## Characterization of $\Gamma _f$

We prove Theorem 1.5 in the following two lemmas. First, we observe that Lemma 3.4 quickly yields the first part of Theorem 1.5.

### Lemma 4.1

Suppose that $f :{{\mathbb {B}}}_n \rightarrow {{\mathbb {B}}}_N$ is a rational proper map in normal form and *f* is not linear. Then $\Gamma _f \le U(n)$ is a subgroup that is defined by a single invariant polynomial.

### Proof

Let $f=\frac{p}{g}$ and apply Lemma 3.4. In particular, if $\rho (z,{\bar{z}}) = |{g(z)} |^2-\Vert {p(z)} \Vert ^2$, then the lemma implies that

13 $$\begin{aligned} \Gamma _f = \bigl \{ U \in U(n) : \rho (Uz,{\overline{Uz}}) = \rho (z,{\bar{z}}) \bigr \} . \end{aligned}$$

$$\square$$

We now prove the second part of Theorem 1.5.

### Lemma 4.2

Given any group $\Gamma \le U(n)$ that is defined by finitely many invariant polynomials, there exists a polynomial proper map $f :{{\mathbb {B}}}_n \rightarrow {{\mathbb {B}}}_N$ such that $f(0)=0$ and $\Gamma _f = \Gamma$.

We note that the *N* required depends on the inviariant polynomials given.

### Proof

Suppose that $\Gamma \le U(n)$ is the group given by the invariant polynomials $\rho _1,\ldots ,\rho _\ell$:

14 $$\begin{aligned} \Gamma = \bigl \{ U \in U(n) : \rho _j(Uz,{\overline{Uz}}) = \rho _j(z,{\bar{z}}), j=1,\ldots ,\ell \bigr \} . \end{aligned}$$

We can ensure that $\rho _j(0)=0$ for each *j*. We will construct a single polynomial that defines $\Gamma$. Suppose that $\rho _j$ is of bidegree $(d_j,d_j)$. Write $k_j = d_1+d_2+\cdots +d_j$ and construct

15 $$\begin{aligned} \rho (z,{\bar{z}}) = \Vert {z} \Vert ^2 \sum _{j=1}^\ell \Vert {z} \Vert ^{2k_j} \rho _j(z,{\bar{z}}) . \end{aligned}$$

Note that $\Vert {Uz} \Vert ^{2} = \Vert {z} \Vert ^2$ for any unitary *U*, and that each polynomial $\Vert {z} \Vert ^{2k_j} \rho _j(z,{\bar{z}})$ only has monomials of degree $2k_{j-1}+1$ through $2_{k_j}$. In particular, no two $\Vert {z} \Vert ^{2k_j} \rho _j(z,{\bar{z}})$ have monomials of the same degree. Therefore, $\rho (Uz,{\overline{Uz}}) = \rho (z,{\bar{z}})$ if and only if $\rho _j(Uz,{\overline{Uz}}) = \rho _j(z,{\bar{z}})$ for each *j*. The first factor $\Vert {z} \Vert ^2$ ensures that $\rho$ has no holomorphic or antiholomorphic terms. Suppose that $\rho$ is of bidegree (*d*, *d*). Consider

16 $$\begin{aligned} R(z,{\bar{z}}) = \sum _{j=1}^d \frac{1}{d} \Vert {z} \Vert ^{2j} . \end{aligned}$$

We have that $R(z,{\bar{z}})=1$ when $\Vert {z} \Vert ^2=1$ and moreover its matrix of coefficients is positive definite as it is a sum of squares of holomorphic polynomials. These polynomials thus give a polynomial proper map of balls. For a small $\epsilon > 0$ write

17 $$\begin{aligned} R_\epsilon (z,{\bar{z}}) = R(z,{\bar{z}}) + \epsilon \rho (z,{\bar{z}}) (1-\Vert {z} \Vert ^2) . \end{aligned}$$

Note that the matrix of coefficients of $R_\epsilon$ has zeros at all the entries for holomorphic or antiholomorphic terms. Moreover, except for the constant, the diagonal terms of the matrix for *R* are positive and at least $\frac{1}{d}$. Thus, for a small enough $\epsilon$, the matrix for $R_\epsilon$ is still positive definite and hence a sum of squares of holomorphic polynomials

18 $$\begin{aligned} R_\epsilon (z,{\bar{z}}) = |{f_1(z)} |^2+\cdots +|{f_N(z)} |^2 . \end{aligned}$$

Since the matrix of coefficients is zero at the entries for holomorphic and antiholomorphic terms, the polynomials $f_j$ can be picked so that $f_j(0)=0$ for all *j*. As $R_\epsilon = 1$ when $\Vert {z} \Vert =1$, we have a proper map of balls $f=(f_1,\ldots ,f_N)$. We have that $U \in \Gamma _f$ if and only if

19 $$\begin{aligned} R_\epsilon (Uz,{\overline{Uz}})= & {} R(Uz,{\overline{Uz}}) + \epsilon \rho (Uz,{\overline{Uz}}) (1-\Vert {Uz} \Vert ^2) \nonumber \\= & {} R(z,{\overline{z}}) + \epsilon \rho (Uz,{\overline{Uz}}) (1-\Vert {z} \Vert ^2) = R_\epsilon (z,{\bar{z}}). \end{aligned}$$

That is, $U \in \Gamma _f$ if and only if $\rho (Uz,{\overline{Uz}})=\rho (z,{\bar{z}})$ and that defines $\Gamma$. The map *f* is the desired map. $\square$

## Constructions

The following basic result is useful for constructing maps with a given denominator. The result we prove below is a straightforward construction that gives a numerator of a specific degree, for denominators close to 1. There is a far deeper result, see D’Angelo [12] or Catlin-D’Angelo [5], that any polynomial that does not vanish on the closed ball is a denominator of a proper map of balls. In that case however, the degree cannot be bounded.

### Proposition 5.1

Given any polynomial $G :{{\mathbb {C}}}^n \rightarrow {{\mathbb {C}}}$ of degree $d-1$ such that $G(0)=0$, there exists an $\epsilon > 0$, $N \in {{\mathbb {N}}}$, and a polynomial $P :{{\mathbb {C}}}^n \rightarrow {{\mathbb {C}}}^N$ of degree *d* such that $P(0)=0$ and $\frac{P}{1+\epsilon G}$ is a proper map of ${{\mathbb {B}}}_n$ to ${{\mathbb {B}}}_N$. The *N* can be taken to be one less than the number of different monomials of degree *d* or less in *n* variables.

A proof for $d=3$ was given in [1] and must be somewhat modified for higher degree.

### Remark 5.2

It may be convenient in some constructions to consider $1+G(\epsilon z)$ instead of $1+\epsilon G(z)$, and the proof follows in exactly the same way. That is, given an affine variety, there is a proper map with this variety as the pole set provided we can we can dilate it sufficiently far away from the origin.

### Proof

One starts with the polynomial

20 $$\begin{aligned} R(z,{\bar{z}}) = \sum _{j=1}^d \frac{1}{d} \Vert {z} \Vert ^{2j} . \end{aligned}$$

If we ignore the row and column corresponding to purely holomorphic and purely antiholomorphic terms (which is a single row and column), its matrix of coefficients is positive definite. Set *N* to be the rank of this matrix, which is one less than the number of all holomorphic monomials in *n* variables of degree *d* or less. Moreover $R=1$ when $\Vert {z} \Vert =1$. Now consider

21 $$\begin{aligned} r(z,{\bar{z}}) = \epsilon \bigl (G(z)+\overline{G(z)}\bigr )\bigl (1-\Vert {z} \Vert ^2\bigr ) -R(z,{\bar{z}}) + 1. \end{aligned}$$

The rank of this matrix is $N+1$, as we will have a 1 on the coefficient of the matrix for the constant term, the off diagonal elements are all small if $\epsilon > 0$ is small, and all other diagonal elements are negative and of size roughly $\frac{1}{d}$ or larger (assuming $\epsilon$ is small). Thus, for a small $\epsilon$, the matrix of coefficient will have 1 positive and *N* negative eigenvalues. The matrix of coefficients for

22 $$\begin{aligned} r(z,{\bar{z}})-|{1+\epsilon G(z)} |^2 \end{aligned}$$

has zeros at all the terms corresponding to the pure holomorphic and pure antiholomorphic terms, and hence is of rank *N* and therefore negative semidefinite. In particular, there exists a polynomial map $P :{{\mathbb {C}}}^n \rightarrow {{\mathbb {C}}}^N$ such that

23 $$\begin{aligned} r(z,{\bar{z}}) = |{1+\epsilon G(z)} |^2-\Vert {P(z)} \Vert ^2 . \end{aligned}$$

The degree of *P* is at most *d* as that is the maximal degree of all monomials, and as there were no holomorphic or antiholomorphic terms in the matrix from which we constructed *P*, we can chose *P* to not include the constant monomial, and hence $P(0)=0$. In fact, since $\Vert {1+\epsilon G(z)} \Vert ^2$ has no terms of bidegree (*d*, *d*) and *r* does, we must conclude that *P* is in fact of degree *d* and not less. Since we also get that $r(z,{\bar{z}})=0$ when $\Vert {z} \Vert =1$, we find that $\frac{P}{1+\epsilon G}$ is a proper map.

It is left to show that the map is in lowest terms. If not, that is, if there was a common multiple *h* of the components of *P* and $1+\epsilon G$, then we would have $r(z,{\bar{z}}) = |{h(z)} |^2 A(z,{\bar{z}})$ for some real polynomial *A*. Consider the top degree part of *r*, that is, we look at the bidegree (*d*, *d*) part of *r* which is simply

24 $$\begin{aligned} r(z,{\bar{z}})_{(d,d)} = - \frac{1}{d} \Vert {z} \Vert ^{2d} . \end{aligned}$$

The function *h*, were it nonconstant, would be zero at some points arbitrarily far away from the origin, while $r_{(d,d)}$ and hence *r* must become arbitrarily negative as $\Vert {z} \Vert$ becomes large. So *h* is a constant and $\frac{P}{1+\epsilon G}$ is in lowest terms. $\square$

Using the previous proposition, we can construct a map of degree 4 with denominator

25 $$\begin{aligned} 1+\sigma _1 z_1^2 + \cdots + \sigma _n z_n^2 + \epsilon _1 z_1^3+\cdots +\epsilon _n z_n^3 , \end{aligned}$$

which then necessarily has the trivial group $D_f = \{ I \}$, and hence $\Gamma _f = \{ I \}$.

If the denominator is of the form $1+\sigma _1 z_1^2 + \cdots + \sigma _n z_n^2$, then $D_f = \{ I, -I \}$. If we want $G_f$ to also be $\{ I, -I \}$, then we would need the numerator to be invariant, but that requires degree 4, see Lemma 6.1. For small $\sigma$ we can always construct such a degree 4 map.

### Proposition 5.3

Given $n \in {{\mathbb {N}}}$, there exists an *N* and an $\epsilon > 0$ such that whenever $0< \sigma _1< \cdots< \sigma _n < \epsilon$ then there exists a rational proper map of balls of degree 4 in normal form $f=\frac{p}{g} :{{\mathbb {B}}}_n \rightarrow {{\mathbb {B}}}_N$ (in lowest terms) such that

26 $$\begin{aligned} g(z) = 1 + \sum _{j=1}^n \sigma _j z_j^2 \end{aligned}$$

and such that $p(z)=p(-z)$. In other words, $G_f = \{ I, -I \}$.

We note that the construction in Example 6.2 can be adapted to arbitrary *n* and ensure the existence of such an example whenever $\sigma _1^2+\cdots +\sigma _n^2 < 1$, so $\epsilon = \frac{1}{\sqrt{n}}$ would suffice. The advantage of the approach given here is that it easily generalizes to more complicated denominators.

### Proof

We adapt the proof of Proposition 5.1 from above, however we need to work with forms where only holomorphic and antiholomorphic monomials of even degree arise. Let *N* be the number of monomials in *n* variables of degree 2 and 4. Thus start with

27 $$\begin{aligned} R(z,{\bar{z}}) = \frac{1}{2}\Vert {z} \Vert ^4+\frac{1}{2}\Vert {z} \Vert ^8 . \end{aligned}$$

Again $R=1$ if $\Vert {z} \Vert =1$. Write $G(z) = \sum _{j=1}^n \sigma _j z_j^2$ and construct

28 $$\begin{aligned} r(z,{\bar{z}}) = \bigl ( G(z)+\overline{G(z)}\bigr )\bigl (1-\Vert {z} \Vert ^4\bigr ) -R(z,{\bar{z}}) + 1. \end{aligned}$$

Note the $\Vert {z} \Vert ^4$ in the formula. The matrix of coefficients has nonzero rows and columns only for monomials of even degree, and taking this submatrix we find a full rank matrix of rank $N+1$. As the on diagonal elements are roughly of size $\frac{1}{2}$ or larger as long as $\sigma$s are small, and all off diagonal elements are of size proportional to the $\sigma$s, we find that the number of negative eigenvalues is *N* and there is one positive eigenvalue corresponding the constant 1. The matrix of $|{1+G(z)} |^2$ also only has nonzero rows and columns for the monomials of even degree, and hence so does

29 $$\begin{aligned} r(z,{\bar{z}})-|{1+G(z)} |^2 , \end{aligned}$$

whose matrix again has no elements for the row and column corresponding to the constant. Hence its rank is *N* and it must be a negative semidefinite matrix so there is a polynomial *p*(*z*) only using degree 2 and 4 monomials such that

30 $$\begin{aligned} r(z,{\bar{z}})=|{1+G(z)} |^2 -\Vert {p(z)} \Vert ^2 . \end{aligned}$$

Again $r=0$ on $\Vert {z} \Vert =1$ and we are finished, $\frac{p}{1+G}$ is the desired map. It is in lowest terms by the same argument as in Proposition 5.1. $\square$

## Group invariance of maps with generic $\sigma$

We prove the first part of Corollary 1.4 in the next lemma. The construction part of the corollary we have already done in Proposition 5.3.

### Lemma 6.1

Suppose $f = \frac{p}{g} :{{\mathbb {B}}}_n \rightarrow {{\mathbb {B}}}_N$ is a rational proper map in normal form such that $0< \sigma _1< \ldots < \sigma _n$. Then $G_f$ is either trivial, or $G_f = \{ I, -I \}$, in which case $p(z) = p(-z)$ and $g(z)=g(-z)$. In particular, if $G_f = \{ I, -I \}$ then the degree of *f* is at least 4.

### Proof

First, $G_f \le \Sigma _f$, and as the $\sigma$s are nonzero and distinct, we have that $\Sigma _f$ is composed of diagonal matrices with $\pm 1$ on the diagonal. Via the theorem of Lichtblau [16], $G_f$ must be fixed-point-free and cyclic, and the only such subgroups are $\{ I \}$ or $\{I, -I \}$. Suppose that $G_f = \{ I, -I \}$. As $G_f \le D_f$, we have that $\{I,-I\} \le D_f$ and so $g(z)=g(-z)$. We have

31 $$\begin{aligned} \frac{p(z)}{g(z)} = \frac{p(-z)}{g(-z)} \quad \text {or}\quad p(z)g(-z) = p(-z)g(z) . \end{aligned}$$

As $g(z)=g(-z)$ we find that $p(z)=p(-z)$. Thus all the monomials that appear in *g* and *p* are of even degree. The degree of *p* cannot be 2 as normal form implies that $\deg (g) < \deg (p)$, hence $\deg (p) \ge 4$. $\square$

It is worth remarking that the condition $G_f=\{I, -I\}$ immediately gives that *f* is even, but in the Corollary, we further prove that the numerator and denominator must also be even for ball maps.

The second part of the corollary now follows from Proposition 5.3. The proposition says that the given map satisfies $G_f \ge \{I, -I \}$, and the lemma says that this must be an equality.

Let us give an explicit example in a slightly different way for ${{\mathbb {B}}}_2$. The example is a modification of an example given by Al Helal [19]. An advantage of this construction is that it allows explicit bounds on the $\sigma$’s. On the other hand, it does not generalize easily to more complicated denominators.

### Example 6.2

We will give this example in the $n=2$ case, but it is easy to generalize to higher *n*. We start with an automorphism $\varphi$ of ${{\mathbb {B}}}_3$ as given by (6). We will pick $\alpha = \langle - \sigma _1, 0, - \sigma _2 \rangle$, and as $\alpha \in {{\mathbb {B}}}_3$ we require that $\sigma _1^2+\sigma _2^2 < 1$. Consider the homogeneous proper ball map $H :{{\mathbb {B}}}_2 \rightarrow {{\mathbb {B}}}_3$ given by

32 $$\begin{aligned} H(z_1,z_2) = \bigl (z_1^2,\sqrt{2}\,z_1z_2,z_2^2\bigr ) . \end{aligned}$$

Write $$\varphi \circ H$$ as

33 $$\begin{aligned} \varphi \circ H (z) = \psi (z) = \bigl ( \psi _1(z), \psi _2(z), \psi _3(z) \bigr ) . \end{aligned}$$

Note that the denominator of this map is precisely

34 $$\begin{aligned} 1 + \sigma _1 z_1^2 + \sigma _2 z_2^2 . \end{aligned}$$

The map is not in normal form; while $\psi _2(0)=0$, we have $\psi _1(0) \not = 0$ and $\psi _3(0)\not = 0$. By tensoring $\psi _1$ and $\psi _2$ by *H*, we get a new map that is in normal form; namely, we consider the map

35 $$\begin{aligned} f = \bigl ( ( \psi _1 \oplus \psi _3 ) \otimes H \bigr ) \oplus \psi _2 . \end{aligned}$$

Then $f :{{\mathbb {B}}}_2 \rightarrow {{\mathbb {B}}}_7$ is a degree 4 map in normal form with the desired denominator. The map is invariant under $-I$ as all the monomials that appear are quadratic and hence $G_f = \{ I, -I \}$.
